# Retraction

**DOI:** 10.1080/10717544.2022.2157154

**Published:** 2022-12-28

We, the Editors and Publisher of *Drug Delivery* have retracted the following article:

Sultan Alshehri, Syed Sarim Imam, Afzal Hussain, Mohammad A. Altamimi, Nabil K. Alruwaili, Fahad Alotaibi, Abdullah Alanazi & Faiyaz Shakeel (2020) Potential of solid dispersions to enhance solubility, bioavailability, and therapeutic efficacy of poorly water-soluble drugs: newer formulation techniques, current marketed scenario and patents, *Drug Delivery*, 27:1, 1625–1643, DOI: 10.1080/10717544.2020.1846638

Since publication, concerns have been raised about the similarity of the language, subheadings, and cited examples to a previously published article in *European Journal of Pharmaceutics and Biopharmaceutics:*

Chau Le-Ngoc Vo, Chulhun Park, Beom-Jin Lee (2013) Current trends and future perspectives of solid dispersions containing poorly water-soluble drugs, *European Journal of Pharmaceutics and Biopharmaceutics*, 87:3 Part B, 799-813, DOI: 10.1016/j.ejpb.2013.09.007

When approached for a response to the concerns, the authors were unable to provide a satisfactory response. As this represents a breach of publishing ethics and of warranties made by the author with respect to originality and provenance, we are therefore retracting the article. The corresponding author listed in this publication has been informed.

We have been informed in our decision-making by our policy on publishing ethics and integrity and the COPE guidelines on retractions.

The retraction article will remain online to maintain the scholarly record, but it will be digitally watermarked on each page as ‘Retracted’.

